# The impact of pharmacist-led medication reconciliation and interprofessional ward rounds on drug-related problems at hospital discharge

**DOI:** 10.1007/s11096-022-01496-3

**Published:** 2022-11-03

**Authors:** Helene Studer, Tamara L. Imfeld-Isenegger, Patrick E. Beeler, Marco G. Ceppi, Christoph Rosen, Michael Bodmer, Fabienne Boeni, Kurt E. Hersberger, Markus L. Lampert

**Affiliations:** 1 Pharmaceutical Care Research Group, Department of Pharmaceutical Sciences, University of Basel, Klingelbergstrasse 50, 4056 Basel, Switzerland; 2 Clinical Pharmacy, Institute of Hospital Pharmacy, Solothurner Spitäler AG, Olten, Switzerland; 3 Occupational and Environmental Medicine, Epidemiology, Biostatistics and Prevention Institute, University of Zurich, University Hospital Zurich, Zurich, Switzerland; 4 Hospital Pharmacy, Zuger Kantonsspital AG, Baar, Switzerland; 5 Basel Pharmacoepidemiology Unit, Division of Clinical Pharmacy and Epidemiology, Department of Pharmaceutical Sciences, University of Basel, Basel, Switzerland; 6 Internal Medicine, Zuger Kantonsspital AG, Baar, Switzerland

**Keywords:** Clinical pharmacy, Drug-related problems, Hospital discharge, Medication reconciliation, Medication review

## Abstract

**Background:**

During transitions of care, including hospital discharge, patients are at risk of drug-related problems (DRPs).

**Aim:**

To investigate the impact of pharmacist-led services, specifically medication reconciliation at admission and/or interprofessional ward rounds on the number of DRPs at discharge.

**Method:**

In this retrospective, single-center cohort study, we analyzed routinely collected data of patients discharged from internal medicine wards of a regional Swiss hospital that filled their discharge prescriptions in the hospital’s community pharmacy between June 2016 and May 2019. Patients receiving one of the two or both pharmacist-led services (Study groups: Best Care = both services; MedRec = medication reconciliation at admission; Ward Round = interprofessional ward round), were compared to patients receiving standard care (Standard Care group). Standard care included medication history taken by a physician and regular ward rounds (physicians and nurses). At discharge, pharmacists reviewed discharge prescriptions filled at the hospital’s community pharmacy and documented all DRPs. Multivariable Poisson regression analyzed the independent effects of medication reconciliation and interprofessional ward rounds as single or combined service on the frequency of DRPs.

**Results:**

Overall, 4545 patients with 6072 hospital stays were included in the analysis (Best Care n = 72 hospital stays, MedRec n = 232, Ward Round n = 1262, and Standard Care n = 4506). In 1352 stays (22.3%) one or more DRPs were detected at hospital discharge. The combination of the two pharmacist-led services was associated with statistically significantly less DRPs compared to standard care (relative risk: 0.33; 95% confidence interval: 0.16, 0.65). Pharmacist-led medication reconciliation alone showed a trend towards fewer DRPs (relative risk: 0.75; 95% confidence interval: 0.54, 1.03).

**Conclusion:**

Our results support the implementation of pharmacist-led medication reconciliation at admission in combination with interprofessional ward rounds to reduce the number of DRPs at hospital discharge.

**Supplementary Information:**

The online version contains supplementary material available at 10.1007/s11096-022-01496-3.

## Impact statements

- Pharmacists are able to detect drug-related problems in patients discharged from internal medicine wards, most frequently related to drug choice problems during the hospital stay or at discharge
- The findings of this study support the implementation of the combination of medication reconciliation and interprofessional word rounds to reduce the number of drug-related problems at hospital discharge
- The pharmacist-led medication reconciliation at hospital admission seemed to play a key role in the reduction of the number of drug-related problems at hospital discharge and should therefore be endorsed in daily practice.

## Introduction

Patient safety is at risk at transitions of care, especially at hospital admission and hospital discharge, and it can be impaired by drug-related problems (DRPs) [1, 2]. DRPs are defined as “an event or circumstance involving drug therapy that actually or potentially interferes with desired health outcomes” [3]. A study conducted in Swiss community pharmacies found that frequent DRPs found on discharge prescriptions leading to a pharmaceutical intervention included inappropriate therapy duration, error in the medication process and prescribed drug not available [4].

In order to improve patient safety, different approaches have been developed to reduce DRPs at transitions of care, such as medication reconciliation and involvement of pharmacist during hospital stay (e.g. interprofessional ward rounds). Medication reconciliation is “the formal process in which health care professionals’ [*sic*] partner with patients to ensure accurate and complete medication information transfer at interfaces of care” [5]. This process aims at obtaining a Best Possible Medication History (BPMH) and at reducing unintentional discrepancies [5]. Frequent discrepancies discovered during medication reconciliation include omission of (chronic) medications [6–8], lack of documentation (no clinical document available for medication reconciliation) [7], addition of new medicines [8], and “making a formulary substitution on admission but not switching back to original agent upon patient discharge” [6]. Pharmacist-led medication reconciliation can reduce medication discrepancies at hospital transitions [8, 9].

An additional approach to reduce DRPs in the hospital setting is the involvement of pharmacists in patient care during the hospital stay (e.g. interprofessional ward rounds). This approach showed positive effects on various patient outcomes [10, 11]. In a study conducted in critically ill patients, a significant reduction in mortality and adverse drug events was seen in the intervention group that included a pharmacist in patient care [10]. Other studies in pediatric patients found that pharmacists’ activities, including attending ward rounds, performing educational sessions, being involved in medication safety programs, or reviewing prescriptions in pediatric patients led to a reduction of medication errors [11] and to an improvement of the quality of prescribing [12]. Frequent DRPs identified by clinical pharmacists that led to an immediate change in the patient’s medication regimen during the ward rounds were: inappropriate dose, indicated medicine not prescribed and prescribed medicine not indicated [13].

Although benefits of pharmacist-led services at different points in patient care have been shown, in Switzerland the level of implementation of the two services medication reconciliation at admission and interprofessional ward rounds varies in extent. A national survey conducted in Switzerland revealed that only approximately 10% of hospitals offering clinical pharmacy services, conducted pharmacist-led medication reconciliation at hospital admission regularly (at least weekly). In contrast, interprofessional ward rounds including a pharmacist were regularly (at least weekly) conducted in selected wards in approximately 70% of the hospitals [14]. At hospital discharge, patients are usually given a discharge prescription that can be filled in a community pharmacy, which does not have routine access to clinical data of patients. In contrast, in the cantonal hospital of Zug there is a hospital pharmacy providing clinical pharmacy services to in-patients, and additionally there is a community pharmacy located within the hospital where patients can fill their prescriptions after discharge (from now on referred to as “hospital’s community pharmacy”). The hospital’s community pharmacy opened in February 2016 and has full access to the hospital’s patient records, which allows more advanced pharmacist-led services than in a regular community pharmacy. While the community and the hospital pharmacy are located in separate parts of the hospital, the pharmacists employed, work in both pharmacies. The cantonal hospital of Zug provides an example of a comprehensive pharmacist-led service, where pharmacists are involved throughout the entire hospital stay and documented their activities performed during this service. However, it remains unclear which pharmacist-led service should be integrated throughout the hospitalization process to best support patients at hospitals discharge.

### Aim

The main aim of this study was to analyze the impact of single activities and their combination (pharmacist-led medication reconciliation at admission and interprofessional ward rounds during hospital stay) on the number of DRPs at hospital discharge. The secondary aim was to describe the influence of these pharmacist-led activities on the pattern (type and frequency) of DRPs.

### Ethics approval

The study was approved by the ethics committee of Northwest and Central Switzerland (EKNZ: 2018-01462; 30.08.2018).

## Method

### Study design

In this retrospective, single-center cohort study, we analyzed routinely collected data of inpatients discharged from the cantonal hospital of Zug (180 beds) in Switzerland. The study included adult patients (≥ 18 years) discharged from the internal medicine wards between 1st of June 2016 and 31st of May 2019 (allowing a wash-in phase of at least four months for the different pharmacist-led service) who filled their discharge prescriptions in the hospital’s community pharmacy. Patients were excluded if their DRPs were documented inconclusively (e.g. lack of or insufficient information impeding an interpretation).

The pharmacists of the cantonal hospital of Zug routinely performed structured medication reconciliation in patients admitted to the hospital, conducted interprofessional ward rounds during the hospital stay and performed medication reconciliation followed by medication reviews at hospital discharge.

### Medication reconciliation at admission

At hospital admission, pharmacy technicians, specifically trained in medication reconciliation, regularly took medication histories of most patients with planned admission by using a structured form and at least two different sources of information (e.g. patients own medication, medication history from the general practitioner, patient interview). Subsequently, a pharmacist, specifically trained in medication reconciliation, reviewed the recorded medication and forwarded the resulting BPMH together with potential medication recommendations to the responsible hospital physician. In contrast, for patients with unplanned hospital admissions (emergency admissions), the medication history was taken without a structured form by a physician in the emergency department.

### Interprofessional ward rounds

During the patient’s hospital stay, ward rounds were conducted by physicians and nurses on a daily basis. Once a week, they were accompanied by a clinical pharmacist who focused on identifying and resolving potential and manifest DRPs. Henceforth, the ward rounds accompanied by a pharmacist will be referred to as interprofessional ward rounds.

### Medication reconciliation and medication review at discharge

Prior to hospital discharge, all patients were asked if they agreed to fill their discharge prescriptions in the hospital’s community pharmacy. Pharmacists then performed medication reconciliation (using the discharge prescription and the patients’ own medication) on all discharge prescriptions filled in the hospital’s community pharmacy. In order to detect further DRPs, they screened the discharge prescriptions using predefined risk factors for DRPs and triggers (≥ 5 medicines, ≥ 65 years, lack of instructions on therapy duration, ≥ 1 discrepancy between prescription and home medication, or one of the following medicines: anti-infectives, antiepileptics, oral anticoagulants, antiplatelets) and conducted a medication review based on the number of risk factors/triggers (0–1 risk factors/trigger = simple medication review, 2 risk factors/trigger = intermediate review, ≥ 3 risk factors/trigger = advanced medication review; according to types of medication review defined by the Pharmaceutical Care Network Europe [15]). Pharmacists documented all DRPs discovered in a classification system [16] (adapted GSASA classification system [17]). All patients who filled their prescription in the hospital’s community pharmacy were counselled on their discharge medications by the pharmacy team. Further details on the methods, as well as the types and frequencies of the detected DRPs have been reported elsewhere [16].

**Fig. 1:**
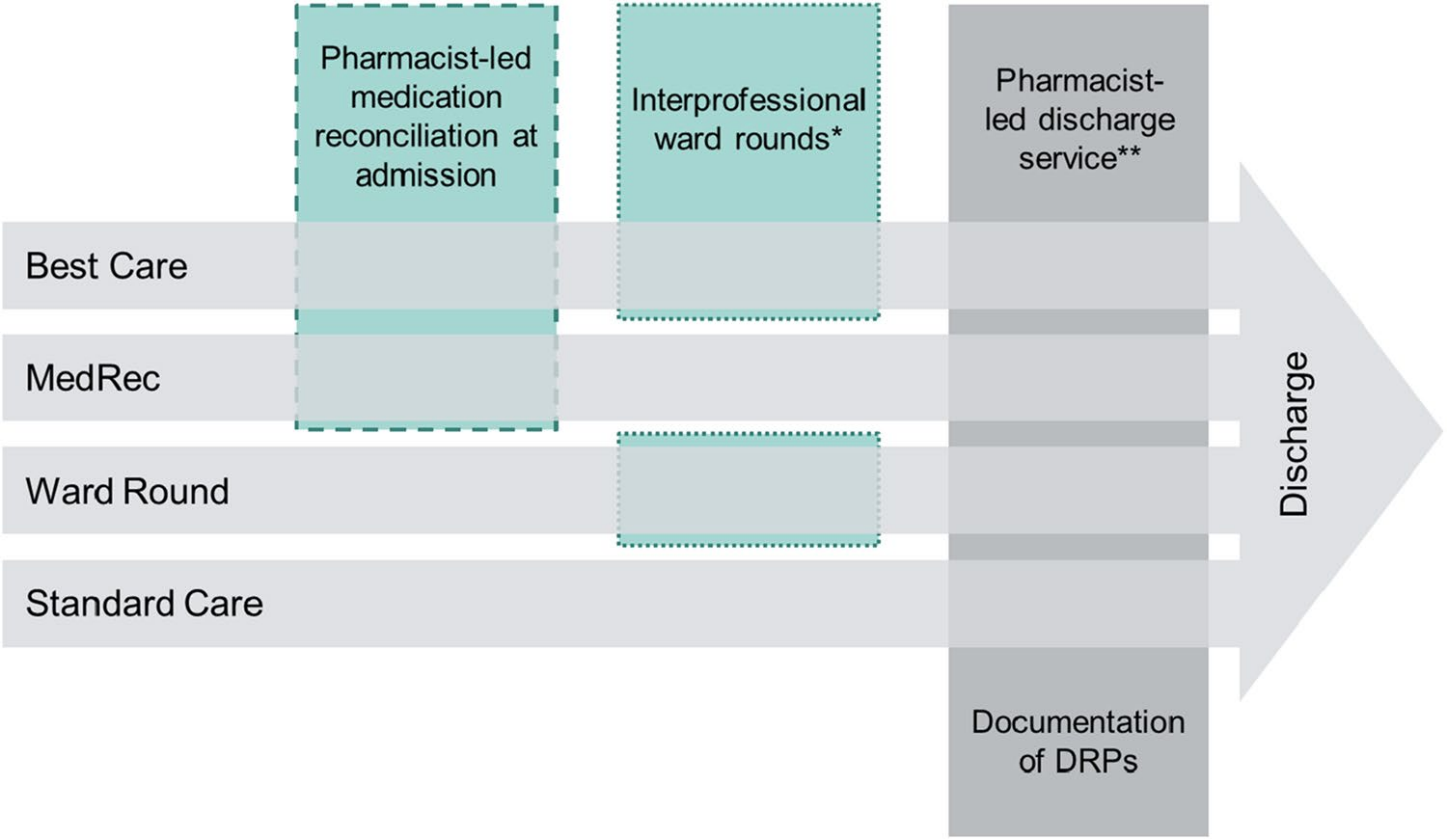
Patient paths at the cantonal hospital of Zug from admission to discharge corresponding to the four defined study groups (dashed border = pharmacist-led service at admission, dotted border = pharmacist-led service during hospital stay). *DRPs* drug-related problems, *MedRec* Medication Reconciliation at admission. *Interprofessional ward rounds = ward rounds including a pharmacist, physician and nurse; **Pharmacist-led discharge service included medication reconciliation at discharge, medication review and discharge counselling

Based on the different exposure to pharmacist-led services, we defined four study groups (Best Care, Medication Reconciliation [MedRec], Ward Round, and Standard Care) (Fig. 1). Patients in the *Best Care group* received pharmacist-led medication reconciliation at admission and interprofessional ward rounds during the hospital stay. In the *MedRec group*, patients received pharmacist-led medication reconciliation at admission and regular ward rounds by physicians and nurses during the hospital stay. In the *Ward Round group*, patients’ medication history at admission was taken by physicians, during the hospital stay they received an interprofessional ward round including a clinical pharmacist. In comparison to these three study groups, patients of the *Standard Care group* had their medication history taken by physicians and they only had standard ward rounds not including a clinical pharmacist. At hospital discharge all 4 groups received pharmacist-led medication reconciliation and medication review.

### Outcomes

The primary outcome of this study was the number of DRPs at hospital discharge in the four study groups. The secondary outcome was the pattern of the causes of DRPs stratified by the four study groups.

### Data analysis

The database used for this analysis was based on information retrieved from the hospital’s patient records, the hospital’s community pharmacy records and the hospital’s clearing office (details shown in Supplement A). For each hospital stay the number of Elixhauser comorbidities [18, 19] was calculated. For the primary outcome, a multivariable Poisson regression model was used with the number of DRPs at discharge as dependent variable and the following independent variables: study group, age at discharge, gender, type of admission, length of stay, number of medicines at discharge and insurance status. The effect sizes were expressed as relative risks (RR) with 95% confidence intervals (CIs). A multivariable logistic regression compared stays without DRPs at hospital discharge to stays with at least one DRP. The independent variables were the same as those mentioned above, and the effect sizes were expressed as odds ratios (OR) with 95% CIs. Distributions of continuous variables are presented as means with standard deviations (SD) if normally distributed, otherwise as medians with interquartile ranges (IQR). For categorical variables, counts and percentages were calculated. Statistical significance was accepted at a *P*-value of ≤ 0.05. The analysis was conducted on hospital stays (one stay corresponded to one discharge prescription). It was possible for patients to have more than one stay during the study period. Analyses were performed using R Version 3.6.1. (R Foundation for Statistical Computing, Vienna, Austria).

Two supplementary models (1) additionally controlling for renal failure and (2) for the number of Elixhauser comorbidities [18, 19] instead of the number of medicines as independent variables were calculated.

## Results

In total, 6087 hospital stays fulfilled the inclusion criteria for this study. Fifteen stays were excluded due to inconclusive documentation of DRPs, leaving 6072 hospital stays of 4545 individual patients for analysis. The distribution of the hospital stays among the study groups was as follows: Best Care n = 72, MedRec n = 232, Ward Round n = 1262, and Standard Care n = 4506. Patient characteristics are presented in Table 1. During the medication reviews at hospital discharge, pharmacists detected a total of 1876 DRPs in the study population. In 1352 (22.3%) hospital stays, at least one DRP was reported. The distribution of the number of DRPs per hospital stay stratified by the four study groups is presented in Table 2.

**Table 1:** Characteristics of hospital stays (n = 6072) overall and stratified by the four study groups: Best Care, MedRec (Medication reconciliation), Ward Round and Standard Care

		Study groups			
	Study population (n = 6072)	Best Care (n = 72)	MedRec (n = 232)	Ward Round (n = 1262)	Standard Care (n = 4506)
Age at discharge, median [IQR]	75 [61, 83]	79 [64, 82]	75 [63, 82]	76 [62, 84]	75 [61, 83]
Female, n (%)	3012 (49.6)	42 (58.3)	134 (57.8)	618 (49.0)	2218 (49.2)
Planned admission, n (%)	592 (9.7)	62 (86.1)	206 (88.8)	66 (5.2)	258 (5.7)
Length of stay (in days), median [IQR]	4.6 [2.9, 7.5]	6.15 [3.15, 8.12]	3.2 [2.1, 6.1]	6.65 [4.1, 10.1]	4.1 [2.7, 6.8]
Number of medicines at admission, median [IQR]	5 [3, 9]	7 [4, 11]	7.5 [5, 11]	6 [3, 10]	5 [2, 9]
Number of medicines at discharge, median [IQR]	7 [4, 10]	8 [6, 12]	8 [5, 11]	8 [5, 11]	7 [4, 10]
Number of Elixhauser comorbidities [18, 19], median [IQR]	2 [1, 4]	3 [2, 5]	2 [1, 4]	3 [2, 4]	2 [1, 4]
Frequency of specific Elixhauser comorbidities^a^, n (%)					
Hypertension (uncomplicated and complicated)	3065 (50.5)	45 (62.5)	111 (47.8)	672 (53.2)	2237 (49.6)
Cardiac arrhythmias	1565 (25.8)	19 (26.4)	43 (18.5)	325 (25.8)	1178 (26.1)
Renal failure	1529 (25.2)	21 (29.2)	50 (21.6)	374 (29.6)	1084 (24.1)
Fluid and electrolyte disorders	1181 (19.4)	12 (16.7)	25 (10.8)	323 (25.6)	821 (18.2)
Diabetes (uncomplicated and complicated)	1070 (17.6)	17 (23.6)	35 (15.1)	271 (21.5)	747 (16.6)
Congestive heart failure	984 (16.2)	14 (19.4)	24 (10.3)	242 (19.2)	704 (15.6)
Chronic pulmonary disease	736 (12.1)	8 (11.1)	29 (12.5)	193 (15.3)	506 (11.2)
Cancer	695 (11.4)	12 (16.7)	43 (18.5)	143 (11.3)	497 (11.0)
Health insurance status, n (%)					
Standard	4412 (72.7)	72 (100.0)	149 (64.2)	1251 (99.1)	2940 (65.2)
Semi-private	29 (0.5)	0 (0.0)	3 (1.3)	0 (0.0)	26 (0.6)
Private	1631 (26.9)	0 (0.0)	80 (34.5)	11 (0.9)	1540 (34.2)

^a^Elixhauser comorbidities occurring in more than 10% of patients in the overall study population

**Table 2:** Distribution of the number of drug-related problems (DRPs) at hospital discharge per stay stratified by the four study groups (n = 6072 hospital stays)

		Study groups			
	Study population (n = 6072)	Best Care (n = 72)	MedRec (n = 232)	Ward Round (n = 1262)	Standard Care (n = 4506)
Number of stays without DRP at discharge, n (%)	4720 (77.7)	64 (88.9)	189 (81.5)	956 (75.8)	3511 (77.9)
Number of stays with at least one DRP, n (%)	1,352 (22.3%)	8 (11.1)	43 (18.5)	306 (24.2)	995 (22.1)
Number of DRPs at discharge per stay, n (%)					
1	956 (15.7)	7 (9.7)	28 (12.1)	215 (17.0)	706 (15.7)
2	301 (5.0)	1 (1.4)	14 (6.0)	70 (5.5)	216 (4.8)
3	67 (1.1)	0 (0.0)	1 (0.4)	17 (1.3)	49 (1.1)
4	21 (0.3)	0 (0.0)	0 (0.0)	3 (0.2)	18 (0.4)
5	4 (0.1)	0 (0.0)	0 (0.0)	0 (0.0)	4 (0.1)
6	3 (0.0)	0 (0.0)	0 (0.0)	1 (0.1)	2 (0.0)

*MedRec* Medication reconciliation

### Primary outcome

The Poisson regression model and the logistic regression model both showed a statistically significant association with fewer DRPs and no DRPs, respectively, at hospital discharge in the Best Care group (Table 3). The MedRec group showed a substantial trend, regarding the Poisson model nearly reaching statistical significance in comparison to the Standard Care group. Each additional medicine was associated with a 10%-increase in the relative risk for more DRPs at discharge, and each additional year of patient age increased the relative risk by 2%. We ran two sensitivity analyses, to check whether our results would substantially differ (1) when we included the number of medicines at admission instead of the number of medicines at discharge as an independent variable and (2) when we included the number of medicines at admission instead of the number of medicines at discharge plus the difference between the number of medicines at admission and at discharge as independent variables. Both sensitivity analyses showed nearly identical results (not shown).

**Table 3:** Poisson regression model with the number of drug-related problems (DRPs) as outcome, and logistic regression model for the stays with no versus at least one DRP, n = 6072 stays

	Poisson regression model for the number of DRPs at discharge	Logistic regression model for the number stays with no versus at least one DRP at discharge
	Relative risk (95% CI)	Odds ratio (95% CI)
**Study group, Standard Care**	**1.00 [Reference]**	**1.00 [Reference]**
**Study group, Best Care**	**0.33 (0.16, 0.65)**	**0.37 (0.17, 0.82)**
**Study group, MedRec**	**0.75 (0.54, 1.03)**	**0.78 (0.51, 1.19)**
**Study group, Ward Round**	**0.96 (0.85, 1.08)**	**1.00 (0.85, 1.18)**
Age, per additional year	1.02 (1.02, 1.02)	1.02 (1.01, 1.02)
Sex, male	1.00 [Reference]	1.00 [Reference]
Sex, female	0.96 (0.88, 1.05)	0.91 (0.80, 1.03)
Admission type, emergency	1.00 [Reference]	1.00 [Reference]
Admissions type, planned	0.99 (0.80, 1.22)	0.90 (0.68, 1.19)
Length of stay, per additional day	1.00 (0.99, 1.01)	1.00 (0.99, 1.01)
Number of medicines at discharge, per additional medicine	1.10 (1.09, 1.11)	1.12 (1.10, 1.13)
Insurance status, standard	1.00 [Reference]	1.00 [Reference]
Insurance status, half-private	0.67 (0.32,1.44)	0.64 (0.23,1.78)
Insurance status, private	0.97 (0.87, 1.08)	1.00 (0.86, 1.16)

*DRP* drug-related problem, *CI* confidence interval, *MedRec* Medication Reconciliation, *bold* study groups

Two further models (1) controlling for renal failure and (2) for the number of Elixhauser comorbidities [18, 19] instead of the number of medicines as independent variables, both showed similar associations (Supplement B and C, respectively). In the model controlling for renal failure the Poisson regression model showed a RR of 1.11 (95% CI 1.00, 1.22). The model including the number of Elixhauser comorbidities instead of the number of medicines as independent variable showed a RR of 1.09 (95% CI 1.06, 1.12) in the Poisson regression model.

### Secondary outcome

**Fig. 2:**
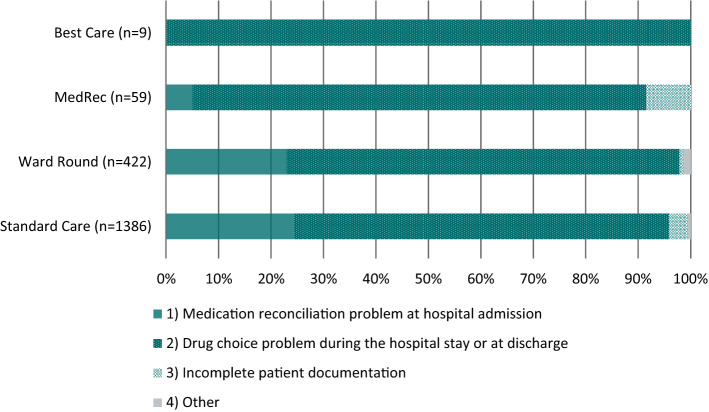
Causes of drug-related problems within the four study groups, shown as percentages (n = 1876 DRPs); *MedRec* medication reconciliation

On the discharge prescriptions of the 6072 hospital stays, pharmacists detected a total of 1876 DRPs (mean = 0.31 DRPs per hospital stay) during medication reconciliation and medication review. The analysis of the causes of these DRPs (Fig. 2) showed that in the Best Care group none of the DRPs were caused by medication reconciliation problems (incorrect medication recorded, omission of a medication, incorrect strength/dose recorded) at hospital admission, while in the MedRec group three (5.1%) were caused by medication reconciliation problems, 97 (23.0%) in the Ward Round group and 338 (24.4%) in the Standard Care group.

## Discussion

In this retrospective single-center cohort study, the aim was to investigate the impact of pharmacist-led services, specifically medication reconciliation at admission and/or interprofessional ward rounds on the number of DRPs at discharge. We found a significant association of the combined pharmacist-led services (medication reconciliation at admission and interprofessional ward round during hospital stay) with less DRPs at hospital discharge in comparison to Standard Care. Pharmacist-led medication reconciliation at admission as a single service showed a trend to having less DRPs at hospital discharge. Both these groups (Best Care and MedRec) received a structured pharmacist-led medication reconciliation at hospital admission, while in the Ward Round and Standard Care group the medication history was taken by the hospital physicians.

These findings are in line with the literature. Medication reconciliation at transitions of care was effective in identifying medication discrepancies [20, 21], and in reducing unintentional medication discrepancies [21]. The combination of medication reconciliation and patient education at discharge reduced readmissions [6]. Undetected medication discrepancies at hospital admission often result in persisting discrepancies during the hospital stay and even until hospital discharge [22]. Even though some of these medication discrepancies might be of negligible clinical significance during the hospital stay, their clinical importance likely increases if they persist after hospital discharge [23]. Studies found that 21–42% of the discrepancies detected in patients’ medications at admission were judged to be clinically relevant, a few were rated as serious or life-threatening [20, 21].

Regarding the study group that only received interprofessional ward rounds, our findings did not show less DRPs at hospital discharge compared to the Standard Care group. We believe that pharmacists’ interventions during ward rounds focus more on patient’s acute health problems than on the optimization of discharge prescriptions. Furthermore, if pharmacists’ interventions during the ward rounds are based on a medication list that is not accurate, they may not be able to detect and resolve issues that are due to a medication reconciliation problem at admission. In the Ward Round group, approximately a quarter of DRPs were related to medication reconciliation problems at admission. In the present study, we did not evaluate the effect of interprofessional ward rounds on DRPs *during* the hospital stay. But other studies that included pharmacists in ward rounds or multidisciplinary teams, had shown an improved quality of medication prescribing [12] and a reduction in adverse events and mortality [10].

We found associations between the number of DRPs and other independent variables included in the regression models, namely the number of medicines at discharge, age, renal function and comorbidities. But out of these well-known risk factors [24, 25] only the number of medicines at discharge can be influenced by pharmacists [24]. We observed an association per additional medicine at discharge. Therefore, checking for opportunities for deprescribing should be promoted.

In our study, medication reconciliation and obtaining the best possible medication history at hospital admission was undertaken by pharmacy technicians under the supervision of clinical pharmacists. Other studies have successfully involved pharmacy technicians in the medication reconciliation process [6, 26]. Involving properly trained pharmacy technicians in medication reconciliation can help to free pharmacist resources for clinical tasks such as subsequent medication reviews. It can also help to implement the process at a lower cost [27]. Implementation of a risk score at admission to select patients that most benefit from subsequent pharmacist-led interventions might also help optimize the use of limited resources [28].

Concerning the frequency and the pattern of DRPs at hospital discharge, we found that pharmacists were able to identify a total of 1876 DRPs, resulting in one of three discharge prescriptions with at least one DRP. Most of the DRPs were caused by prescribing problems during the hospital stay or at discharge. Comparing the whole study population with the Best Care group reveals 50% less patients with one or more DRPs at hospital discharge (22.3% compared to 11.1%). A study from New Zealand found a similar rate as we discovered in the overall study population, with a frequency of 25% of discharge prescriptions with at least one DRP [29]. However, since the studies used different documentation tools for DRPs, this comparison has to be interpreted with caution. Furthermore, in our study, the data collection was part of the routine discharge process and it was not especially collected for study purpose, which might have led to an under-reporting of DRPs, as some minor problems of low clinical relevance may not always have been documented as DRPs.

Overall, our study further confirms the benefits of pharmacist-led services at transitions of care. As observed in the regression models, medication reconciliation at admission as single activity was associated with a trend to less DRPs at discharge and interprofessional ward rounds were not, we hypothesize that medication reconciliation plays a key role in the reduction of the number of DRPs. Conducting systematic medication reconciliation at transitions of care is also endorsed by the Swiss Patient Safety Foundation and international recommendations of the World Health Organization and the International Pharmaceutical Federation. They recognize the risk that medication discrepancies pose to medication safety during transitions of care and advocate the involvement of pharmacists in this process and an enhanced transfer of information from the hospital to the primary care providers [30–32].

### Strengths and limitations

The strengths of this study were the large sample size with over 6000 hospital stays and the fact that the study was conducted with routinely collected real-life data. The investigated services were not implemented for the purpose of the study, but were part of the routine process, which highlights the feasibility of the services. The limitations of our study should be taken into account when interpreting our results. First, this was a single center study, which limits the generalizability of the findings. Second, the sample sizes of the study groups differed substantially. However, despite the small size of the Best Care group, the results still showed statistically significantly less DRPs at hospital discharge compared to the Standard Care group. Further, as patients could have more than one hospital stay during the study period, the first hospital stay may have influenced the number of DRPs of later hospital stays.

## Conclusion

The findings of this study showed that pharmacists frequently detected DRPs at hospital discharge. Moreover, a combination of pharmacist-led medication reconciliation at hospital admission instead of medication history taken by physicians and interprofessional ward rounds during the stay showed an independent significant association with a lower number of DRPs on the discharge prescription. The pharmacist-led medication reconciliation at hospital admission seemed to play a key role for a lower number of DRPs, as it showed a substantial trend for less DRPs as a single activity. These results should stimulate the inclusion of pharmacists in the patients’ paths and clinicians’ workflows throughout the hospital stay, especially at transitions of care, to improve patient safety.

## Supplementary Information

Below is the link to the electronic supplementary material.

Supplementary file 1 (PDF 616 kb)
