# Supplementary material for: The impact of pharmacist-led medication reconciliation and interprofessional ward rounds on drug-related problems at hospital discharge
Source: Int J Clin Pharm. 2022 Nov 3;45(1):117–25. doi: 10.1007/s11096-022-01496-3 (PMC9938815; doi:10.1007/s11096-022-01496-3)
Supplement: Supplementary file 1 — (PDF 616 kb) [file 11096_2022_1496_MOESM1_ESM.pdf]

The impact of pharmacist-led medication reconciliation and interprofessional ward rounds on drug-related problems at hospital discharge

AUTHORS

Helene Studer [1,2]*, Tamara L. Imfeld-Isenegger [1], Patrick E. Beeler [3], Marco G. Ceppi [4,5], Christoph Rosen [4], Michael Bodmer [6], Fabienne Boeni [1,2], Kurt E. Hersberger [1], Markus L. Lampert [1,2]

*corresponding author:

Helene Studer

University of Basel

Pharmaceutical Care Research Group

Klingelbergstrasse 50

4056 Basel

Switzerland

helene.studer@unibas.ch

16-digit ORCID: 0000-0001-9186-8822

Affiliations

[1] Pharmaceutical Care Research Group, Department of Pharmaceutical Sciences, University of Basel, Basel, Switzerland

[2] Clinical Pharmacy, Institute of Hospital Pharmacy, Solothurner Spitäler AG, Olten, Switzerland

[3] Occupational and Environmental Medicine, Epidemiology, Biostatistics and Prevention Institute, University of Zurich & University Hospital Zurich, Zurich, Switzerland

[4] Hospital Pharmacy, Zuger Kantonsspital AG, Baar, Switzerland

[5] Basel Pharmacoepidemiology Unit, Division of Clinical Pharmacy and Epidemiology, Department of Pharmaceutical Sciences, University of Basel, Basel, Switzerland

[6] Internal Medicine, Zuger Kantonsspital AG, Baar, Switzerland

SUPPLEMENT

Supplement A: Development and validation of the database

The database used for this analysis was based on information retrieved from the hospital's patient records, hospital's community pharmacy records and hospital's clearing office. The overview below shows the information that was extracted from the records and the information used for the linkage.

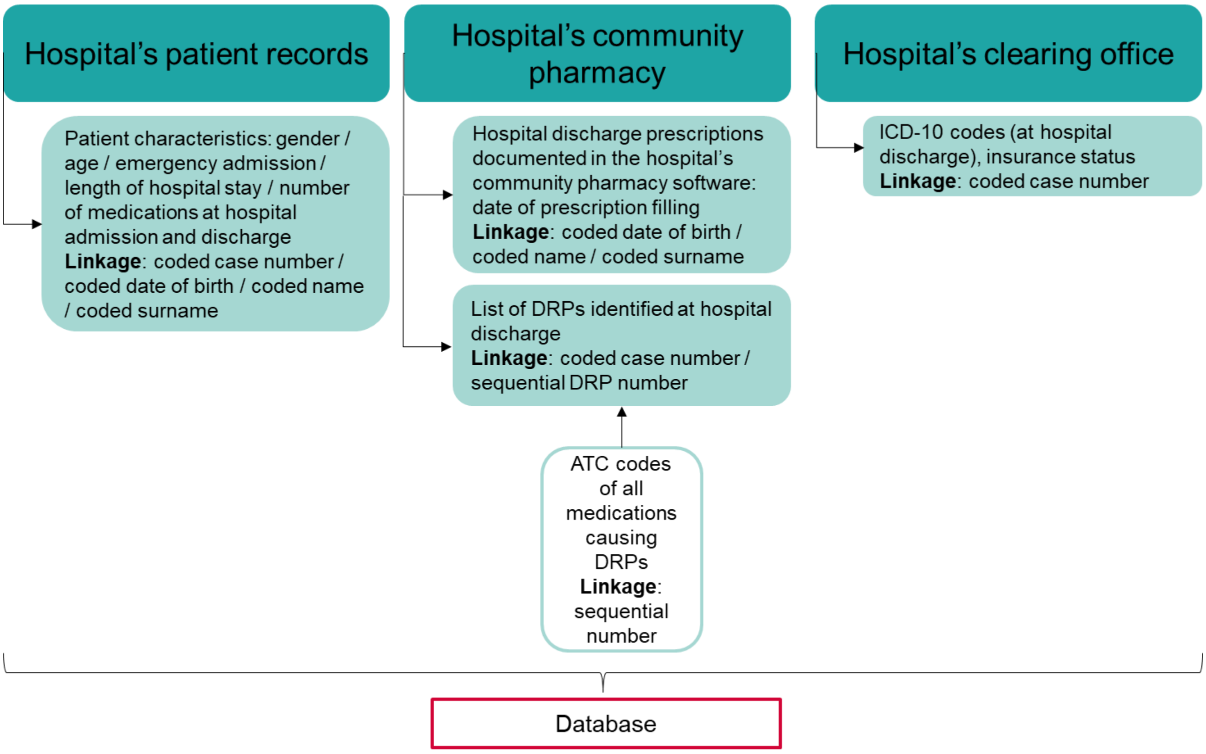

The output tables of database used for the analyses were validated by two research pharmacists of the study team, who had temporary reading access to the records. For the validation a random sample of 1% of patient stays was selected.

Supplement B: Poisson regression model and logistic regression model additionally including renal failure as independent variable (n=6072 stays)

	Poisson regression model for the number of DRPs at discharge	Logistic regression model for the number stays with no or at least one DRP at discharge
	Relative risk (95% CI)	Odds ratio (95% CI)
**Study group, Standard Care**	**1.00 [Reference]**	**1.00 [Reference]**
**Study group, Best Care**	**0.33 (0.17, 0.65)**	**0.37 (0.17,0.82)**
**Study group, MedRec**	**0.75 (0.55, 1.04)**	**0.78 (0.51, 1.20)**
**Study group, Ward Round**	**0.96 (0.85, 1.08)**	**1.00 (0.85, 1.18)**
Age, per additional year	1.02 (1.01, 1.02)	1.02 (1.01, 1.02)
Sex, male	1.00 [Reference]	1.00 [Reference]
Sex, female	0.96 (0.88, 1.06)	0.91 (0.81, 1.04)
Admission type, emergency	1.00 [Reference]	1.00 [Reference]
Admissions type, planned	0.99 (0.80, 1.22)	0.90 (0.67, 1.19)
Length of stay, per additional day	1.00 (0.99, 1.01)	1.00 (0.99, 1.01)
Number of medicines at discharge, per additional medicine	1.10 (1.09, 1.11)	1.11 (1.10, 1.13)
Insurance status, standard	1.00 [Reference]	1.00 [Reference]
Insurance status, half-private	0.69 (0.33, 1.47)	0.66 (0.24, 1.85)
Insurance status, private	0.97 (0.87, 1.08)	1.01 (0.87, 1.17)
Renal failure	1.11 (1.00, 1.22)	1.21 (1.05, 1.40)

CI = confidence interval, DRP = drug-related problem, MedRec = Medication Reconciliation, bold = study groups

Supplement C: Poisson regression model and logistic regression model including the number of Elixhauser comorbidities per patient (instead of the number of medicines) as independent variable (n=6072 stays)

	Poisson regression model for the number of DRPs at discharge	Logistic regression model for the number stays with no or at least one DRP at discharge
	Relative risk (95% CI)	Odds ratio (95% CI)
**Study group, Standard Care**	**1.00 [Reference]**	**1.00 [Reference]**
**Study group, Best Care**	**0.35 (0.18,0.69)**	**0.39 (0.18,0.86)**
**Study group, MedRec**	**0.82 (0.59,1.12)**	**0.84 (0.55,1.28)**
**Study group, Ward Round**	**0.97 (0.87,1.10)**	**1.02 (0.86,1.20)**
Age, per additional year	1.02 (1.02,1.02)	1.02 (1.02,1.03)
Sex, male	1.00 [Reference]	1.00 [Reference]
Sex, female	1.00 (0.91,1.09)	0.95 (0.84,1.07)
Admission type, emergency	1.00 [Reference]	1.00 [Reference]
Admissions type, planned	1.02 (0.83,1.26)	0.95 (0.72,1.25)
Length of stay, per additional day	1.01 (1.00,1.02)	1.01 (1.00,1.02)
Insurance status, standard	1.00 [Reference]	1.00 [Reference]
Insurance status, half-private	0.74 (0.35,1.58)	0.70 (0.26,1.89)
Insurance status, private	0.98 (0.88,1.10)	1.01 (0.88,1.18)
Number of Elixhauser comorbidities per patient	1.09 (1.06,1.12)	1.11 (1.07,1.15)

CI = confidence interval, DRP = drug-related problem, MedRec = Medication Reconciliation, bold = study groups
